# Verbal learning in the context of background music: no influence of vocals and instrumentals on verbal learning

**DOI:** 10.1186/1744-9081-10-10

**Published:** 2014-03-26

**Authors:** Lutz Jäncke, Eliane Brügger, Moritz Brummer, Stephanie Scherrer, Nsreen Alahmadi

**Affiliations:** 1 Psychological Institute, Department of Neuropsychology, University of Zurich, Zurich, Switzerland; 2 Program of Higher Educational Studies, Department of Special Education, King Abdulaziz University, Jeddah, 21589, Saudi Arabia; 3 University of Zurich, Binzmühlestrasse 14, Zurich, 8050, Switzerland

## Abstract

**Background:**

Whether listening to background music enhances verbal learning performance is still a matter of dispute. In this study we investigated the influence of vocal and instrumental background music on verbal learning.

**Methods:**

226 subjects were randomly assigned to one of five groups (one control group and 4 experimental groups). All participants were exposed to a verbal learning task. One group served as control group while the 4 further groups served as experimental groups. The control group learned without background music while the 4 experimental groups were exposed to vocal or instrumental musical pieces during learning with different subjective intensity and valence. Thus, we employed 4 music listening conditions (vocal music with high intensity: VOC_HIGH, vocal music with low intensity: VOC_LOW, instrumental music with high intensity: INST_HIGH, instrumental music with low intensity: INST_LOW) and one control condition (CONT) during which the subjects learned the word lists. Since it turned out that the high and low intensity groups did not differ in terms of the rated intensity during the main experiment these groups were lumped together. Thus, we worked with 3 groups: one control group and two groups, which were exposed to background music (vocal and instrumental) during verbal learning. As dependent variable, the number of learned words was used. Here we measured immediate recall during five learning sessions (recall 1 – recall 5) and delayed recall for 15 minutes (recall 6) and 14 days (recall 7) after the last learning session.

**Results:**

Verbal learning improved during the first 5 recall sessions without any strong difference between the control and experimental groups. Also the delayed recalls were similar for the three groups. There was only a trend for attenuated verbal learning for the group passively listened to vocals. This learning attenuation diminished during the following learning sessions.

**Conclusions:**

The exposure to vocal or instrumental background music during encoding did not influence verbal learning. We suggest that the participants are easily able to cope with this background stimulation by ignoring this information channel in order to focus on the verbal learning task.

## Introduction

It is a popular believe that background music during learning exerts beneficial effects on learning. For example, a modern internet platform provides playlists claiming to improve learning and mental focus (https://play.spotify.com/album/3NOgHfjdYZQLdTsBZYhNrZ). The idea that listening to background music boosts learning has a long tradition and has especially been proposed by Georgi Lozanov, a Bulgarian psychotherapist [1] who developed a teaching method (*suggestopedia*) in which background music (mostly classical music) during learning plays a pivotal role. In a 1993 review 10 studies were summarized [2] supporting this view. Nine of the reviewed studies used classical music as background stimulation (mostly baroque music which has been suggested by Lazanov to be the most efficient learning enhancer).

Besides these studies, which have been designed in the context of Lozanov’s suggestopedia, several further studies without any relation to the suggestopedia school have been conducted examining the influence of background music on learning. For example, when the text to be learned is sung instead of being spoken recall of these text passages is much better [3]. Language learning (especially learning a second language, L2) has been shown to benefit from music listening during learning or when the learning material has been transformed into sung melodies [4]. This has recently been replicated in a study during which a second language (here Mandarin) has been learned with accompanying music [5]. In this study, individuals who learned Chinese performed better on all tests examining the learning progress. However, this positive influence of music on learning a second language was only evident for the group learning Mandarin but not for a group learning Arabic. However, the allocation of the subjects to the Chinese and Arabic groups was not random, thus some cohort effects might had some influence here.

A recent paper has focused on the role of background music on memory consolidation [6]. The authors identified that listening to arousing music (irrespective of the experienced valence of the presented music) during memory consolidation improved memory performance. This effect has been attributed to a kind of general neurophysiological arousal associated with the depletion of glucocorticoids and catecholamines enhancing memory consolidation. A further paper supported this view in demonstrating that listening to relaxing music during memory consolidation reduces memory performance [7] thus supporting the view that neurophysiological arousal is beneficial for consolidation.

However, negative or non-existing effects have also been reported. For example Salame and Baddeley [8] reported that listening to background vocal music during encoding interferes with verbal learning and results in reduced memory performance. A more recent study of our group studied the influence of auditory background melodies on verbal learning and identified no influence on recall performance [9]. However, the simultaneously recorded EEG revealed that background music increases cortical activation, most likely indicating increased cortical (and cognitive) effort to inhibit and down-regulate the interfering melodies to achieve good performance in verbal learning. Thus, this study supports the view that although there might be no difference in the behavioral measures of verbal learning there are however, neurophysiological indices indicating the increased effort for learning while simultaneous background music stimulation was present. In some way this finding supports the wealth of studies supporting the view that background music mostly acts as a distraction to the primary tasks [10,11].

In this experiment we are interested in readdressing the question whether background music might have an effect on verbal learning. Based on our first experiment in which we found no effect of background stimulation on verbal learning [9] we redesigned our experimental design. First of all we now use real music and not as in the first experiment artificial tone sequences. Second we examined a larger sample, and thirdly, we studied how learning performance changes during the course of repeated learning in the context of background stimuli. It might be possible that background music exerts its negative (or positive) influence at different stages of learning. For example, background music could be more disturbing at the beginning of learning and the learner might adapt to the background music after a while. In addition, we are interested in studying whether vocals and instrumental music might influence verbal learning differently. Since the primary task is to learn words, vocal music might interfere more strongly with the encoding and recall of verbal material than instrumental music.

## Methods

### Participants

226 participants (133 women and 93 men) were recruited for this experiment. They were invited to take part in a learning experiment through flyers distributed around campuses of the UZH and the ETH, an internet webpage of the UZH and the ETH, online social networking, and word-of-mouth. All subjects underwent an evaluation to screen for chronic diseases, mental disorders, medication, drug or alcohol abuse, and were tested with different neuropsychological and psychological tests (for measuring mental rotation ability, attention, and psychometric intelligence). After this screening 199 subjects (133 women and 66 men) were subjected to the final statistical analysis. 27 subjects were excluded because of excessive drug intake or neurological or psychiatric disorders. In addition, the subjects completed a questionnaire asking for music preferences, how often they listened to music in especially during learning sessions, and which music genre and which particular musical pieces they prefer. In addition, all subjects indicated for how long they played a musical instrument. And how often they had practiced their instrument. According to this variable four groups were defined with one group never have played and practiced an instrument (no practice – P-: n = 35), a second group, which indicated to have practiced for on average 6.8 yrs (few practice – P+: n = 67), a third group with 9.5 yrs of musical practice (moderate practice – P++: n = 32), and finally a group, which has practiced quite a lot with on average 13 yrs (frequent practice – P+++: n = 27). This variable (musical practice: PRAC) was used as control variable for the statistical analysis. Normal hearing ability was confirmed for all subjects using standard audiometry. For intelligence assessment, a short test [12,13] was used that is known to correlate with standard intelligence test batteries (r = 0.7 - 0.8). In addition, the NEO-FFI [14] was used to measure the personality trait “extraversion” because of its strong correlation with dual task performance [15,16]. All subjects were consistently right-handed, as assessed with the Annett-Handedness-Questionnaire [17]. All subjects indicated not having received formal musical education for more than five years during their school years and that they had not played any musical instrument in the last 5 years. We also asked the subjects whether they had previously learned while listening to music. Most of them confirmed having done so. Each subject gave informed consent and received 30 Swiss Francs for participation. The study was carried out in accordance with the Declaration of Helsinki principles and was approved by the ethics committee of the University of Zurich as part of a larger research project on music and cognition.

### Study design

The basic principle of this study was to explore verbal memory performance under different music background stimulation conditions similar to a previous experiment of our group [9]. The subjects repeatedly performed a verbal learning test while musical pieces were presented during verbal encoding and recall. Verbal learning was examined using words from a standard verbal learning test, which is frequently used for neuropsychological examinations with German-speaking subjects (*Verbaler Lerntest*, VLT). This test has been shown to validly measure verbal short and long-term memory [18,19]. This test was slightly modified for the needs of this experiment. In this experiment we used 50 German words, with 30 words taken from the original verbal learning test. We included 20 new words to prevent ceiling effects. Thus, this test material was identical to the test material we have used in a previous experiment [20]. During encoding these 50 words were randomly presented for 3 seconds each via PowerPoint and a beamer to a screen in front of the subjects (font = Calibri; font size = 96; color = black, distance from subject to screen 2–3 m). The subjects were instructed to look at the words attentively and to learn them by heart. Those subjects learning during music stimulation were not specifically instructed how to cope with the background music. After the encoding phase (duration = 150 seconds) the subjects were instructed to write down all remembered words on an answer sheet placed in front of them (recall phase; duration = 4 minutes). After the recall phase a new trial started. This procedure was repeated 4 times (trial1 – trial4) yielding 4 recall scores (RECALL1 - RECALL4). During these 4 phases subjects of the music background groups received music stimulation. After the 4th trial a break of 10 minutes was included followed by a further recall test (RECALL5). The 6th recall (RECALL6) followed 30 minutes after the 5th recall. Approximately 2 weeks (on average 13.4 days) after the 6th recall a long-term delayed recall test was performed (RECALL7). The recall tests 5, 6 and 7 were all conducted without any music stimulation even for the music listening groups. The tests were conducted as group tests with 4–8 subjects participating simultaneously at each session. The music stimuli were presented via wireless headphones (Sennheiser HDR 130).

### Musical stimuli and group allocation

Contrary to the previous study of our group we have used real musical pieces, which have been rated as emotionally positive. Our intention was to use positive music with high and low experienced intensity since the study of Judde et al. [6] has shown that the subjectively experienced intensity exerts strong influences on memory performance especially on memory consolidation. Furthermore, we were interested in testing whether verbal learning is influenced differently by simultaneously listening to vocals or to instrumental music. Thus, we also used vocal and instrumental music, resulting in 4 music listening conditions (vocal music with high intensity: VOC_HIGH, vocal music with low intensity: VOC_LOW, instrumental music with high intensity: INST_HIGH, instrumental music with low intensity: INST_LOW) and one control condition (CONT) during which the subjects learned the word lists in silence. All subjects were randomly assigned to one of these five experimental groups. These five groups did not differ significantly in terms of age, extraversion/introversion, attention performance, mental rotation performance, or educational level (all variables tested with Kruskal-Wallis-U-test). However, there was marginally significant differences for IQ (p = 0.057), with subjects from the INST group demonstrating a slightly higher IQ. Therefore IQ was used as a covariate for the statistical analysis.

The musical pieces were collected on the basis of a pilot test during which 50 subjects (mostly university students from the UZH and ETH) evaluated 31 music pieces from a collection of modern music frequently presented in radio programs or which have been used in previous experiments [6,21]. These musical pieces were combined into a playlist using the internet platform http://www.grooveshark.com and sent to these subjects via e-mail. The subjects were asked to rate these musical pieces with respect to the experienced valence and intensity using a 1–9 Likert scales for intensity and valence (intensity: “1” = not at all arousing, “9” = very strongly arousing; valence: “1” = not at all liking, “9” = very strongly liking). Based on these ratings we selected 19 music pieces, which were rated at least as very positive (with a value of >5 on the valence scale). In addition, we selected musical pieces rated as arousing (with a value > 5) and less arousing (value < 4.8). Furthermore, we chose vocal and instrumental music. The chosen music pieces are listed in Table 1. These music pieces were also rated for valence and intensity during the main experiment. Contrary to the pilot experiment the music pieces, which have been rated as evoking low and high intensity did not differ in terms of the subjectively experienced intensity. Thus, we decided to combine the experimental groups receiving low and high intensity musical pieces during encoding and recall into one group. Thus, for the final analysis we worked with three groups: vocal (VOCAL) and instrumental (INST) music as well as the control group (CONT). The sample characteristics of the three groups are depicted in Table 2.

**Table 1:** Used music with average rated valence and intensity

	**Duration (s)**	**Valence**	**Intensity**
***Vocal music (strong subjective intensity)***			
(Peter Fox) Alles Neu	258	5.38	5.07
(Die Toten Hosen) Bonnie und Clyde	211	5.79	5.38
(Die Fantastischen Vier) Troy	210	5.33	5.58
(Die Ärzte) Junge	188	5.00	5.80
(Xavier Naidoo) Sie sieht mich nicht	267	6.47	5.26
***Vocal music (weak subjective intensity)***			
(Gisbert zu Knyphausen) Spieglein, Spieglein	160	5.21	4.69
(Die Söhne Mannheims) IzOn	298	5.14	4.55
(Die Fantastischen Vier) Tag am Meer	255	5.04	3.89
(Freundeskreis) Anna	366	5.38	4.72
(Wir sind Helden) Ode an die Arbeit	223	5.41	4.24
***Instrumental (strong subjective intensity)***			
(Howard Show) Anduril	159	5.40	5.10
(Holst) The Planets - Jupiter, the Bringer	480	6.42	6.55
(Alvfen) Midsommarvaka	540	5.46	5.14
(Klaus Badelt) The Medallion Calls	112	5.50	5.80
***Instrumental (weak subjective intensity)***			
(John Williams) Nocturnal Activities	360	5.40	3.90
(Bill Conti) Fanfare for Rocky	155	5.20	4.60
(John Williams) Scherzo for Motorcycle and Orchestra	169	5.30	4.40
(John Williams and William Ross) Reunion of Friends	300	5.70	4.40
(New Worlds Orchestra) Many Meetings – Soundtrack of Lord of the Rings	194	5.40	4.80

(intensity: “1” = not at all arousing, “9” = very strongly arousing; valence: “1” = not at all liking, “9” = very strongly liking).

**Table 2:** Mean sample characteristics of the three groups studied

	**Control**	**Vocal**	**Instrumental**
Age	25.6 ± 5.9	25.23 ± 5.43	26.65 ± 6.76
Education (1: low 5 high)	3.4 ± 1	3.5 ± 1	3.5 ± 0.9
IQ	100.2 ± 10.7	101.9 ± 11.9	105.7 ± 14.0
Extraversion/Introversion	2.75 ± 0.44	2.55 ± 0.52	2.58 ± 0.53
N of subjects	40	79	80
% female	67.5 %	65.8 %	67.5 %

### Statistical analysis

**Figure 1:**
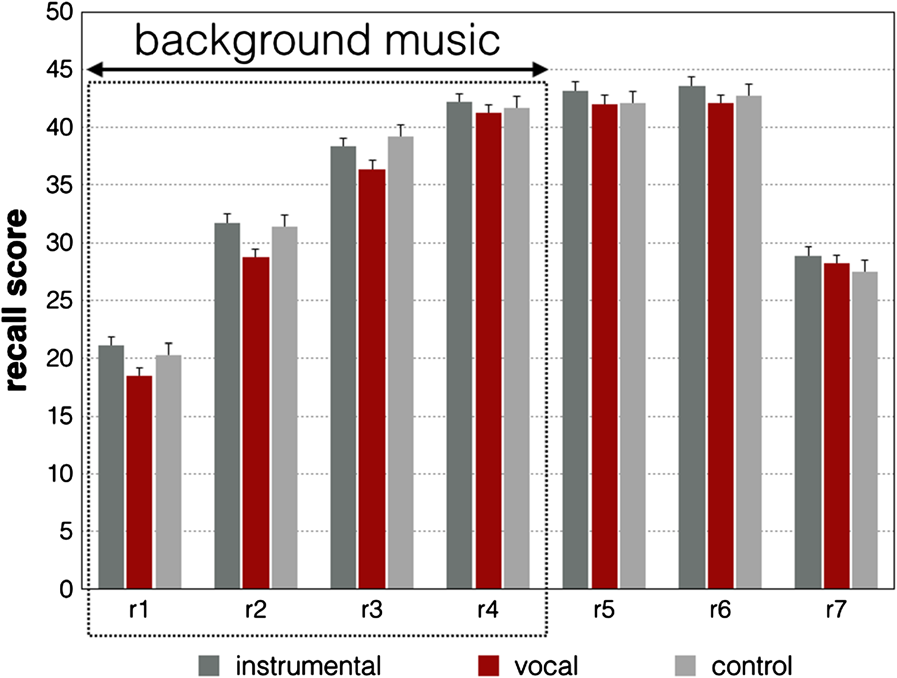
**Average recall performance (and standard errors of the mean) for the 7 recall stages (r1-r7) broken down for the three groups (control, vocal, and instrumental music).** Recall 5 (r5) and recall 6 (r6) were conducted 10 and 30 minutes after recall 4 (r4). Recall 7 (r7) was conducted 2 weeks after the experiment. The participants of the vocal and instrumental groups received background music stimulation during the learning sessions r1-r4. The recall scores presented here are adjusted for psychometric intelligence.

For the recall tests the number of correctly recalled words was calculated for each recall trial resulting in 7 recall measures for each group. Thus, we obtained three learning curves, one for each group. The mean recall scores for each group are depicted in Figure 1. These learning curves were subjected to a repeated measures ANCOVA with IQ as covariate. For the repeated measurements factor and the interaction including the repeated measurements factor we used the multivariate variant to cope with heteroscedasticity of variances [22]. In addition, we also performed a multivariate one-way MANCOVA for all recall measures with IQ as covariate to compare the recall performance of the three groups separately for each recall measure. In case of heterogeneity of variances we used the Brown-Forsyth correction. A p of < =0.05 was defined as significant. Besides the p values we also calculated effect size measures since it is important to quantify the effect independent of sample size. Here we used eta^2^.

## Results

As already mentioned above, the three groups did not differ in terms of age, education, mental rotation performance, attention performance, gender, and musical experience (all p values at least > 0.15). However, there was a marginally significant difference between the groups for IQ (F(2,196) = 3.025, p = 0.051). Thus, we used IQ as covariate for the following statistical analyses.

First, we calculated a two-way repeated measures ANCOVA with *Time* as repeated measurements factor (recall1 – recall7) and *Group* as grouping factor (CONT, VOC, INST). For testing the repeated measurements factor we used the MANCOVA approach to guard against heteroscedasticity. For the MANCOVA F-test we used Wilk’s lambda. For *Time* we received a significant effect (F(6,190) = 12.994, p <0.001, eta^2^ = 0.291) representing the fact that the recall score improves from trial 1 to 7. The interaction between *Time* and *Group* was not significant (F(12,382) = 1.43, p = 0.150, eta^2^ = 0.043). There was no general *Group* difference (F(2,195) = 1.39, p = 0.252, eta^2^ = 0.014). Planned trend analyses conducted within this ANCOVA revealed strong linear (F(1,195) = 8.64, p = 0.004, eta^2^ = 0.042) and quadratic trends (F(1,195) = 52.685, p < 0.001, eta^2^ = 0.213) for the learning curves with only one significant interaction with *Group* for the linear trend (F(2,195) = 3.043, p = 0.05, eta^2^ = 0.03). Graphical inspection of the learning curves revealed that the subjects of the VOCAL group showed lower recall scores for the first three learning sessions while the recall scores in sessions 4, 5, 6 and 7 seemed to be similar to the recall scores of the other two groups. Thus, we performed a further MANOVA with every recall score (recall1 – recall7) as dependent variable and compared the VOCAL group with the other groups ((INST + CONT)/2). This MANCOVA revealed a trend for a significant multivariate between-groups difference (F(7,198) = 1.94, p = 0.064, eta^2^ = 0.067). The subsequently performed ANOVAs for the single recall scores (recall1 – recall7) with the planned comparisons between the VOCAL group and the other groups revealed small differences between these groups for the first three recall scores (recall1: p = 0.025, eta^2^ = 0.025; recall2: p = 0.019, eta^2^ = 0.03; recall3: p = 0.045, eta^2^ = 0.02).

## Discussion

This study is the second study of a series of experiments in which we examine the influence of background music on learning and in particular on verbal learning. In the first experiment of this series we used artificial melodies to control for individual differences in musical preference and for memory effects [9]. Using these stimuli we identified no influence of musical background on verbal learning. However, a critical aspect of this first study is the fact that the musical pieces we used were artificial and did not evoke strong emotions. Therefore, we designed the present study for which we employed “real” music and also used vocals as well as instrumentals to test whether these types of music exert different influences on verbal learning. A further point we tried to realize was to keep learning as natural as possible. Thus, we tested the subjects in groups (similar to a classroom setting) and continuously recorded their learning progress. In addition, we tried to separate immediate from delayed learning performance. After doing this, we identified no significant influence of background music on verbal learning. This non-detectable influence was evident for the immediate recall tests as well as for the delayed recall tests. It is worth mentioning that there was also no influence on late recall measured 14 days after learning.

Interestingly, there was no specific influence of the particular type of music on learning since vocals or instrumental music did not differ in terms of their non-detectable influence on learning. This is particularly important because we hypothesized that listening to vocals during learning would interfere especially with encoding, consolidation, and recall of verbal material. However, there was no strong and statistically significant influence of listening to vocals on verbal learning.

There was a small (non-significant) effect of listening to vocals upon verbal learning. The subjects who have been exposed to vocals demonstrated reduced recall in the first three recall sessions at the beginning of the experiment. This slight decrease in recall performance at the beginning disappeared throughout the experiment. This small effect might be due to an initially present interference effect of vocals on verbal learning and resembles the effect reported by Salame and Baddeley [8]. Salame and Baddeley tested verbal recall in the presence of vocal and instrumental music and identified that those subjects who heard vocal music performed worse than the subjects in a silent condition. This kind of verbal interference was quite small in our experiment and the subjects were able to efficiently cope with this interference, and they could do so even better and more efficiently at the end of the experiment. In our previous experiment we also obtained brain activation measures during learning and we identified that “demanding” background music (musical pieces which were fast and out-of-tune) was associated with increased brain activation. Obviously, the subjects could cope with detracting information by increasing the neurophysiological activation of the involved brain areas.

It is worth mentioning that there was even no positive and enhancing effect on verbal learning, an effect, which has been proposed by several researchers and theoreticians. For example it has been proposed that music would activate the brain thus evoking supporting chemical reactions (e.g., depletion of glucocorticoids and catecholamines) [6,7].

A possible reason for the non-existing influence of background music on verbal learning could be that verbal learning of this material was too easy. If the background music was too easy it could be not disturbing enough in order to interfere with verbal learning. If we would have used more demanding verbal stimuli (e.g., words in a foreign language) it might have been possible that more processing resources would have been devoted to control encoding, consolidation, and recall. Thus, background music might have been more interfering in this situation, like a kind of dual task, with background music as the secondary task and verbal learning as the primary task.

A further reason for the non-existing influence of background music could be that the subjects focused their attention strongly on the learning task. When doing this they literally ignored the background music. From neurophysiological studies it is known that ignoring external stimuli strongly attenuates activity in those brain areas and neural networks processing the ignored stimuli [23-25]. Thus, it could be that the background music is not processed that intensively, causing no strong neurophysiological and psychological interference. Possibly, if we would have asked the subjects to listen attentively to the music or to perform some kind of discrimination tasks with the musical pieces this would have exerted detrimental effects. Gopher and Donchin [26] have demonstrated that the amount of processing resources allocated to different information channels is most important for the influence of the secondary task on the processing of the primary task. The more processing resources are allocated to the secondary task the worse is the performance of the primary task. In the context of our experiment this implies that our subjects have spent more resources on the primary task (verbal learning) than on the secondary task (listening to the music).

However, a final question is still unanswered. As mentioned in the introduction there are some quite influential ideas appearing in the literature proposing that background music positively influences learning in general [1]. With our experiment we cannot support these views, at least not with the music used and in the context of our experimental paradigm. Despite the fact that we have used pleasant and arousing music, we did not detect positive influences on verbal learning. Maybe arousing music supports learning only when it is presented 15–20 minutes after encoding of the learning material (and not during encoding as in our experiment). Or it could be that background music only exerts beneficial effects on learning when the subjects are under-activated, tired, less aroused, or their memory systems operate inefficiently due to neurological handicaps. In fact some studies have shown that background noise (not music) can enhance cognitive performance in inattentive participants [27]. Background stimulation might enhance arousal and diminishes drowsiness in these patients, which might also improve cognitive performance.

### Limitations

A methodological limitation of this study is that the participants listened to music that they didn’t choose by themselves. Normally, when learning we choose the background music which we believe is most suitable for us to support learning. Thus, we will consciously or unconsciously choose the music which we believe would best fit our needs. We also choose how long and how often we listen to background music while learning, depending on our mood and/or attentional level. Thus, we decide when we listen to background music and which musical piece is running as background music. In our experiment this was not controlled for. However, future experiments should clarify whether these aspects might have influences on the effects of background music on cognition in general and learning in particular.

## Conclusion

Using pleasant and arousing vocal and instrumental background music we found no strong influence of background music on verbal learning. We suggest that the participants are easily able to cope with this background stimulation by ignoring this information channel in order to focus on the verbal learning task.

## Competing interests

The authors declare that they have no competing interests.

## Authors’ contributions

LJ designed the experimental paradigm, performed the statistical analysis and drafted the manuscript. AN, EB, MB, and SS reviewed the statistical analysis and drafted the manuscript. All authors read and approved the final manuscript.
